# Determinants of inter birth interval among married women living in rural pastoral communities of southern Ethiopia: a case control study

**DOI:** 10.1186/1471-2393-13-116

**Published:** 2013-05-20

**Authors:** Zenebu Begna, Sahilu Assegid, Wondwosen Kassahun, Mulusew Gerbaba

**Affiliations:** 1 Department of Epidemiology, Jimma University, College of Public Health and Medical Sciences, Jimma 378, Ethiopia; 2 Department of Population and Family Health, Jimma University, College of Public Health and Medical Sciences, Jimma 378, Ethiopia

## Abstract

**Background:**

Though birth interval has beneficial effects on health status of the mother and their children, it is affected by range of factors some of which are rooted in social and cultural norms and the reproductive behaviors of individual women. However, there was limited data showed the determinants of birth intervals in rural pastoral communities of South Ethiopia. Therefore, the study was aimed to assess the determinants of inter birth interval among women’s of child bearing age in Yaballo Woreda, Borena zone, Oromia Regional State, Ethiopia.

**Methods:**

A community based unmatched case–control study with multi stage sampling technique was conducted from January to March 2012. Cases were women with two subsequent birth intervals of less than three years and controls were women with two subsequent birth intervals between three and above years. Simple random sampling technique was employed to select six hundred fifty two (326 cases and 326 controls) study subjects. All explanatory variables that were associated with the outcome variable (birth interval) during bivariate analysis were included in the final logistic model. Multivariable backward logistic regression when P values less than or equal to 0.05 and 95% CI were used to determine independent determinants for the outcome of interest.

**Results:**

The median duration of birth interval was 31 & 40 months among cases and controls respectively. Variables such as number of children (AOR 3.73 95% CI: (1.50, 9.25), use of modern contraceptives (AOR 5.91 95% CI: (4.02, 8.69), mothers’ educational status (AOR 1.89 95% CI: (1.15, 3.37), and sex of the child (AOR 1.72 95% CI: (1.17, 2.52) were significantly associated with birth intervals.

**Conclusions:**

Concerted efforts to encourage modern contraceptive use, women education, and breastfeeding should be made.

## Background

Ethiopia, one of the populous nations in Africa, has a total population of more than 80 million with annual population growth rate of 2.6% [1]. According to Ethiopian demographic and health survey (2011), total fertility rate (TFR) was 4.8 which is substantially higher among rural women than among urban women where rural women give birth to nearly three more children during their reproductive years than urban women (5.5 and 2.6, respectively) [2].

Fertility is one of the important principal components of population dynamics that determine the size and structure of the population of a given country. According to Bogart’s fertility determinants factors affecting fertility are broadly classified into proximate (direct) and distal (indirect) factors [3-5].

Birth interval is the length of time between two successive live births [2]. The length of the birth interval is dependent on the duration of each component, with the postpartum amenorrhea and the menstruating intervals having greater variability in their duration than the other [6,7]. Optimal spacing between pregnancies has greater health advantages for both mother and child, which can give an opportunity for the mother to recover from pregnancy, labor and lactation [8-10]. Longer time period between births allows the next pregnancy and birth to occur more likely to be at full gestation and growth [11,12]. Studies also revealed optimum birth interval can improve the health status of children. Survival of children could increase each year if all women had optimal birth interval and decrease child mortality [13-18].

Empirical evidence from many different cultural settings has identified several correlates of birth intervals including breast feeding, contraceptive use, and maternal education [13-15,19-23]. However, to the investigators knowledge there is limited evidence and there is no study conducted so far to assess factors that determine birth intervals among pastoral communities setting of southern Ethiopia. Therefore, analysis of factors that can influence birth interval among women will provide local and regional planners useful information that could encourage optimal intervals.

## Methods

### Study setting and period

The study was conducted in Yaballo Woreda, one of the fourteen Woreda’s, found in Borena zone of Oromia region, at the southeast of Ethiopia from January to March 2012. It is located 576 km away from Addis Ababa. Yaballo Woreda has 23 kebeles and according to the national census of 2007, the total population of the Woreda in 2011/12 is projected to be 96,697. Of this 51,250 of them are females. The Woreda consists of 21,370 women in child bearing age. More than 95% of the populations are pastoralist [1]. There are four health centers, sixteen health posts, five private clinics, and one non-governmental organization working on reproductive health interventions in the area. Fifty six rural health extension workers were found in the community.

### Study design and populations

A community based unmatched case control study design was employed. The source populations for the study were all women of child bearing age who experienced at least two successive deliveries and the last delivery within the last five years prior to the data collection found in the Woreda. Study populations were randomly selected women of child bearing age who experienced at least two successive deliveries with history of short birth intervals (the birth interval of less than 3 years between two successive births) for cases and selected women of child bearing age who experienced at least two successive deliveries with history of optimum birth intervals (the birth interval 3–5 years between two successive births including 3 and 5) for controls. Clients who were seriously ill and couldn’t communicate, mother of history of twins, index child mothers who had history of abortion between the last two successive births were excluded from the study.

### Sample size and sampling

The sample size was determined by the formula used for unmatched case control study using Open EPI INFO version 3 .5.1.software. Assumptions used to estimate the sample size were the proportion of control with contraceptive use 29% [2] , minimum detectable of odds ratio of 2, at 5% level of precision, power of 80%, with one to one ratio among cases and controls, considering 5% non-response rate and design effect of 2, the final sample size became 652 (326 each). To get the study subjects two stage sampling techniques was used to do census of women of child bearing age among randomly selected kebeles of the Yaballo Woreda before the actual data collection process was implemented. First seven kebeles from twenty three kebeles of Yaballo Woreda were chosen randomly.

Then each household from the seven kebeles was visited and the total family size was registered. For those households with women of child bearing age having minimum of two deliveries and at least her last delivery was within the last five years prior to the study period was identified and corresponding house identification number was given to develop sampling frame. Based on the above techniques a total of 561 cases and 530 controls were identified and eligible for the study. Finally using sampling frame created for each kebeles, simple random sampling technique was employed to select the households that were included in the study subjects defined as cases and controls. Probability to proportional size allocation technique was used in the determination of the number of kebeles and study units included in each kebeles. When two or more women of child bearing age from a household who satisfied the inclusion criteria, lottery method was employed to select one woman from that household.

### Data collection methods

Data were collected by using structured interviewer administered questionnaire which was translated first to Afaan Oromo (language spoken in the study area) by Afaan Oromo speaker who has attended Master of Arts and translated back in to English language by different person who attended Master of Arts in English and literatures at Jimma University to check its consistency. To ensure the quality of data to be gathered from the study subjects, First, data collection instruments were pretested on 5% of the sample out of the selected study kebeles and necessary modifications were made based on the nature of gaps identified in the questionnaire. Seven trained rural health extension workers and four Bsc. nurse graduate whose mother tongue is Afaan Oromo language were recruited for the data collection and supervision respectively. They were trained for two days on procedures of data collection techniques by principal Investigator. On site supervision was carried out during the whole period of data collection on daily basis. At the end of each day questionnaires were reviewed and cross checked for completeness, accuracy and consistency by the principal investigator and corrective measures were under taken.

### Data processing and analysis

Data were edited, coded, entered, cleaned and analyzed using SPSS version 16 software. Frequency distribution was done to check for outliers, consistencies and to identify missing values. Descriptive analysis such as median was computed. Bivariate analysis was performed to identify the association of dependent and independent variables. Odds ratio was computed to see the strength of association between socio-economic, demographic and biological variables and the inter birth intervals. To identify independent predictors, first a bivariate logistic regression was performed (at p<0.25) for each independents and outcome of interest (birth interval). Finally, backward step wise multivariable logistic regression model was done to determine independent predictors of birth intervals where first all measured biological variables and socio demographic variables were included in the model and those variables not significantly associated were eliminated in stepwise followed by re entrance of the left variables until those significant variables were left in the final model. All tests were two-sided and P < 0.05 was considered statistically significant. Ethical clearance was obtained from Health Research and Post Graduate College of Public Health and Medical Sciences Ethical Review Board of Jimma University. Formal letter of permission was written from administrative bodies of the zone, Woreda and kebeles. Letter of cooperation from kebeles administrators was also secured. Finally verbal consent was requested from every study participant included in the study during data collection time after explaining the objectives of the study and the right to withdraw from the study. Confidentiality was also assured.

## Results

### Socio- demographic characteristics

Six hundred thirty six respondents were participated in the study making the response rate of 97.5%. Of this 323(99.0%) of women with short birth interval (cases) and 313(96.0%) of women with optimum birth interval (controls) participated in this study. The median age of women with cases and controls were 30 and 33 years respectively. Most respondents were within the age group 25–29 years which accounts 115(35.6%) for cases and 78(24.9%) for controls. The median age at marriage was 17 years and 16 years for women with cases and control respectively. Most women 315(97.5%) among cases and 291(93.0%) among controls were married. Regarding educational status, 255(78.9%) of cases and 216(69.0%) of controls had no formal education (Table 1).

**Table 1:** Socio-economic, demographic and biological characteristics of study subjects, Yaballo Woreda Borena Zone, Ethiopia, March 2012

**Variables**	**Cases (323)**	**Controls (313)**
	**N (%)**	**N (%)**
**Religion**		
Orthodox	39(12.1)	63(20.1)
Protestant	59(1 8.3)	58(18.5)
Muslim	40(12.1)	30(9.6)
Catholic	10(3.1)	14(4.5)
Wakefata	175(54.2)	148(47.3)
**Ethnicity**		
Oromo	240(74.3)	227(72.5)
Burji	51(15.8)	55(17.6)
Konso	26(8.0)	25(8.0)
Amhara	6(1.9)	6(1.9)
**Maternal Educational**		
No education	255(78.9)	2 16(69.0)
Primary	67(20.7)	86(27.5)
Secondary and above	1(0.3)	11(3.5)
**Husband education**		
No education	215(66.6)	172(55.0)
Primary	101(31.3)	119(38.0)
Secondary and above	7(2.2)	22(7.0)
**Mother occupation**		
House wife	232(71.8)	193(61.7)
Outside home	91(28.2)	120(38.3)
**Husband occupation**		
Husbandry	221(68.4)	225(71.9)
Farmers	20(6.2)	30(9.6)
Merchant	23(7.1)	26(8.3)
Daily worker	42(13.0)	11(3.5)
Others	17(5.3)	21(6.7)
**Age of the mothers**		
20-24	23(7.1)	16(5.1)
25-29	115(35.6)	78(24.9)
30-34	90(27.9)	71(22.7)
35-39	56(17.3)	75(23.9)
40-44	24(7.4)	49(15.7)
45-49	15(4.6)	24(7.7)
**Age at first marriage**		
<18	203(62.8)	206(65.8)
>=18	120(37.2)	107(34.2)
**Sex of index child**		
Male	148(45.8)	201(64.2)
Female	175(54.2)	112(35.8)
**Parity**		
2 children	26(8.0)	32(10.2)
3-4 children	160(49.5)	127(40.6)
>=5 children	137(42.4)	154(49.2)
**Survival status index child**		
Alive	313(96.9)	307(98.1)
Dead	10(3.1)	6(1.9)
**Breast feeding**		
<24 months	320(99.1)	268(85.6)
>=24months	2(0.6)	44(14.1)
**Contraception** use		
Yes	84(26.0)	214(68.4)
No	239(74.0)	99(3 1.6)

• * others include government employ.

### Determinants of inter birth interval

The median duration of birth interval was 31 & 40 months among cases and controls respectively. Women's who had no formal education were 1.9 times more likely to have short birth interval practice as compared to those who had formal education (AOR 1.89, 95% CI (1.15, 3.37)). Mothers whose partners’ were engaged in a daily work were twice times more likely to have short birth interval as compared to those engaged in husbandry (AOR 2.19, 95% CI (1.01, 4.79)).The odds of being exposed in age groups of 35–39 and 40–44 have reduced the chance of having short birth interval by 68% and 78% respectively (AOR 0.32, 95% CI (0.17, 0.60), and 0.22, 95% CI (0.10, 0.49)). Mothers who were not using contraception between their last births were 6 times more likely to experience short birth interval (AOR 5 .91, 95% CI (4.02, 8.69)) (Table 2).

**Table 2:** Predictors of short birth interval, Yaballo Woreda, Borena zone, Ethiopia, March 2012

**Variables**	**COR (95%CI)**	**AOR (95% CI)**
**Mothers education**		
No formal education	1.87(1.12, 3.12)*	1.89(1.15, 3.37)**
Formal education	1	1
**Husband occupation**		
Husbandry	1.00	1.00
Farmers	0.68(0.37, 1.23)	0.49(0.24, 1.00)
Merchant	0.90(0.49, 1.63)	0.72(0.36, 1.43)
Daily worker	3.89(1.95, 7.75)*	2.19(1.01, 4.79)
Others	0.82(0.42, 1.60)	1.17(0.47, 2.89)
**Parity**		
Two children	0.91(0.52, 1.61)	3.73(1.50, 9.25)**
3 & 4 children	1.42(1.02, 1.97)*	2.69(1.23, 5.92)
>=5 children	1.00	1.00
**Sex of the index child**		
Male	1.00	1.00
Female	2.12(1.544, 2.91)	1.72(1.17, 2.52)**
**Age of the mother**		
20-24	0.94(0.48, 1.96)	1.36(0.53, 3.48)
25-29	1.00	1.00
30-34	0.86(0.56, 1.3 1)*	0.68(0.39,1.17)
35-39	0.51(0.32, 0.79)*	0.31(0.17, 0.60)**
40-44	0.33(0.19, 0.59)*	0.22(0.10, 0.49)**
45-49	0.42(0.21, 0.86)*	0.39(0.15, 1.01)**
**Contraceptive use**		
Yes	1.00	1.00
No	6.15(4.36, 8.68)*	5.91(4.02,8.69)**
**Breast feeding**		
<=24 months	26.27(6.31, 109.37)*	30.8 1(6.97,136.19)**
>24 months	1.00	1.00

*Crude Odds Ratios (Significant at P value<0.05 during Bivariate analysis

**Adjusted Odds Ratios (Significant at P value< 0.05 during multivariable logistic regression analysis.

## Discussion

According to this study among socio-economic and demographic factors mothers’ education and husband occupation and age of the mothers, contraceptive use, sex of the index child and breast feeding practices were associated with short birth interval. Maternal education has protective effect for short birth interval practice. This finding is in line with population report of 2002 in 55 sub Saharan countries of Africa which showed women with no education were less likely to space births than educated women. This might be due to the fact that women with more education are more likely to use contraception to prolong their birth intervals and may have access to information as well. In addition, educated women are more likely to be engaged in occupations that are not readily compatible with bearing children. Under this circumstance, therefore, education is expected to lengthen birth intervals [5,11,20]. Sex of the index child was associated with short birth interval. According to result of this finding women’s who had female index child more likely to have short birth interval compared to mothers who had male index child. This is in line with many studies in Africa where women were more likely to have a next child within three years after a birth of a daughter than after a son’s birth [16,20,23].

The study conducted in Southern Ethiopia also showed high proportion of short birth intervals of less than three years (59.8%) follows when the sex of preceding child was female and 55% when the sex of preceding child was male [23] which is comparable with the current study that shows high proportion of short birth intervals of less than three years (61%) follows when the sex of preceding child was female and 42.4% when the sex of preceding child was male. On the contrary, study conducted in Mozambique [8] showed short birth interval practiced when the index child was male. These variations might be due to the differences in sex preference among the different cultural settings. Women who did not use modern contraceptive were more likely to practice short interval length as compared to those who used modern contraceptives (Significant at P value<0.05 during Bivariate analysis). Similar effect of contraceptive use has been observed in a study conducted in Southern and Northern Ethiopia where contraceptive users space birth longer than the non-users in each observed births [5,23].

In the study conducted in Saudi Arabia high proportion of women who had short birth interval (81.9%) breast feed their child less than two years. Of women who breast fed their child greater or equal to two years, high proportion of them had birth interval >35 months[19]. Our study is comparable with the Saudi Arabia’s finding. For example in this study women who breast feed their index child less or equal to two years were more likely to have short birth interval practice as compared to those who breast feed greater than two years. This study is also similar with the study conducted in Mozambique [8] and Southern Ethiopia [23]. In Mozambique 48% of women who did not fed were likely to have intervals less than three years [8]. Similarly in Southern Ethiopia 80% of women who did not breast fed had intervals less than three years [23]. This study could have the following strength; first it is a community based study, used unmatched case control study design which is better to explore the predictors, have high response rate, tried to address the issue of selection bias by excluding women with abortion, and twin index child. But, measuring duration of birth interval with the respondents’ memory since women or their children in the rural setting of the study area have no birth certificates poses recall bias, interviewer bias and the unmatched analysis of the data might underestimate the measure of effect and the quality of data. In addition to this, unusual large OR with large confidence interval also observed in this study which might be due to inadequate sample size to justify the strength of associations. Therefore, readers shall take in to accounts the above limitation when interpreting the finding of this study.

## Conclusion

It was evident that birth interval has beneficial effects for the health of mother and child. But, in this study this birth interval could be affected by many factors such as maternal education, maternal age, and husband’s employment, sex of the index child, breast feeding, and use of modern contraceptives. Interventions made to improve maternal and child health programs should consider the above modifiable factors. For example women education should be encouraged to decrease the likelihood of short birth interval. In addition, promotion of contraceptive use and breast feeding is crucial to promote birth spacing. Awareness raising and cultural promotion of parents should also be made to avoid sex based intervals.

## Competing interests

The authors declare that they have no competing interests.

## Authors’ contributions

ZB, SA, WK & MG participated in all phases of the study including topic selection, design, data collection, data analysis and interpretation. All authors also contribute to write this manuscript. All authors read and approved the final manuscript.

## Pre-publication history

The pre-publication history for this paper can be accessed here:

http://www.biomedcentral.com/1471-2393/13/116/prepub
